# Forecasting Key Retail Performance Indicators Using Interpretable Regression

**DOI:** 10.3390/s21051874

**Published:** 2021-03-08

**Authors:** Belisario Panay, Nelson Baloian, José A. Pino, Sergio Peñafiel, Jonathan Frez, Cristóbal Fuenzalida, Horacio Sanson, Gustavo Zurita

**Affiliations:** 1 Department of Computer Science, Universidad de Chile, Santiago 8370456, Chile; nbaloian@dcc.uchile.cl (N.B.); jpino@dcc.uchile.cl (J.A.P.); spenafie@dcc.uchile.cl (S.P.); crfuenza@dcc.uchile.cl (C.F.); 2 School of Informatics and Telecommunication, Universidad Diego Portales, Santiago 8370190, Chile; jonathan.frez@mail.udp.cl; 3 Allm Inc., Tokyo 150-0002, Japan; horacio@allm.onmicrosoft.com; 4 Department of Information Systems and Management Control, Faculty of Economics and Business, Universidad de Chile, Santiago 8330015, Chile; gzurita@fen.uchile.cl

**Keywords:** Dempster-Shafer Theory, Evidence Regression, supervised learning, retail indicators, foot traffic prediction, time series regression problems

## Abstract

Foot traffic, conversion rate, and total sales during a period of time may be considered to be important indicators of store performance. Forecasting them may allow for business managers plan stores operation in the near future in an efficient way. This work presents a regression method that is able to predict these three indicators based on previous data. The previous data includes values for the indicators in the recent past; therefore, it is a requirement to have gathered them in a suitable manner. The previous data also considers other values that are easily obtained, such as the day of the week and hour of the day of the indicators. The novelty of the approach that is presented here is that it provides a confidence interval for the predicted information and the importance of each parameter for the predicted output values, without additional processing or analysis. Real data gathered by Follow Up, a customer experience company, was used to test the proposed method. The method was tried for making predictions for up to one month in the future. The results of the experiments show that the proposed method has a comparable performance to the best methods proposed in the past that do not provide confidence intervals or parameter rankings. The method obtains RMSE of 0.0713 for foot traffic prediction, 0.0795 for conversion rate forecasting, and 0.0757 for sales prediction.

## 1. Introduction

There are three key indicators to which retail managers pay special attention: foot traffic, which is the number of visitors entering a brick-and-mortar store, conversion rate, which is the ratio of the people who actually make a purchase, and total sales, over a certain period [1]. Predicting the future store performance within a reasonable time frame allows managers to plan the required sales force, in order to provide the necessary stock for dealing with the expected number of customers and sales, and to plan the cash flow. Researchers have developed some models with this goal in the past using analytical as well as data mining-based models.

In the last few years, there has been a trend to pay more attention to models that can not only accurately predict a certain value, but they also give more information regarding the behavior of the model. One additional piece of desirable information that a model may convey is the importance or “weight” (positive or negative) the input variables have in the prediction process. Knowing this, managers can better react to unexpected changes of the environment conditions, such as an extraordinary holiday, or promotion campaigns. Another important information is the degree of confidence or uncertainty of the predicted value, being frequently expressed as the maximum and minimum expected values. These characteristics are often called the “transparency” of the model [2]. A transparent model allows its users to better understand the phenomena they are studying. However, experience has shown that the so called Black-box models, which produce a prediction value without additional information, are more accurate that transparent ones. Thus, the research question of this work is whether it is possible to develop a transparent model for predicting the three key performance indicators for retail stores with an accuracy that was comparable to the black-box methods and providing additional information. In order to address this question, we developed a new model based on Evidential Regression [3], a transparent method that in turn is based on the Dempster–Shafer theory [4], which was extended in order to use k-Nearest Neighbors with a weighted distance. A first version of this model was developed and applied for predicting future health care costs for individuals [5].

Articles reporting on previous works addressing the prediction of the key performance indicators for retail stores include those that achieve interpretability and allow for computing a confidence interval, like the works that were reported in Lam et al. [6] and Arunraj and Ahrens [7]. These ones do not achieve the accuracy that black-box methods do, like the ones reported in Yu et al. [8], Cortez et al. [9], Abrishami et al. [10], and Abrishami and Kumar [11], which achieve high performance, but do not provide interpretability or a confidence interval. All of the previously mentioned works are focused on predicting one indicator. The work reported in [1] does address the prediction of the three indicators at the same time, but it is developed based on analytical methods, with pre-conceived equations, not taking advantage of Data Science, achieving low accuracy and limited interpretability. When considering these past works, we can state that the contribution of our approach is that it produces an output value for the three performance indicators, along with a confidence interval. It also provides a ranking of the input features according to the effect that each one has on the output; features with high values have high impact on the output, which makes the model inherently interpretable.

In order to develop and test the model, we used retail data that were gathered by Follow Up, a customer experience company based in Santiago, Chile. Follow Up has cameras installed at over 3000 stores in several countries, but, for the purpose of experimentation, we used data from some stores that were located in Chile, Colombia, and Peru. Our predictions were made for up to one month in advance. The performance obtained by the prediction method is comparable to the best existing regression models that were reported in the literature. It was considered acceptable by Follow Up and, therefore, it is currently running an implementation of the algorithm to offer a prediction service to its retailing customers. The rest of this paper is organized, as follows: Section 2 presents the related work to the subject of the paper. Section 3 presents the materials and methods. Section 4 describes the experiments that were done with the model and their results. Section 5 presents the discussion. Section 6 presents the conclusions.

## 2. Related Work

Time series forecasting methods can be applied to predict foot traffic, conversion rates, and sales. However, the result will depend on the pedestrian context, which can include variables, such as time of the day, period of the year, geographical location, etc. Previous foot traffic forecasting works have attempted to predict pedestrians’ movements in closed areas or, alternatively, in open areas [12].

For closed areas, one approach is to analyze the possible routes of pedestrians, e.g., [13]. However, this approach requires complete knowledge of the physical environment and the results are probabilistic; they can be translated into a pedestrian forecast only if the incoming visitors number is provided. For open areas, a typically used approach is to place physical sensors on strategic places and analyze the time series data of each one.

Trying to provide performance metrics for retail business, we attempted to predict the transition of pedestrian incoming visitors from an open area to a closed area that is based on data provided by sensors placed at the entrance of each store. This data are combined with sales and other commercial data, such as promotions or discounts.

This research is focused on supervised learning and interpretability of the algorithm, which is the possibility to track the decisions made by machine [14]. Supervised learning [15] is a machine learning technique that trains an algorithm or statistical model through examples. Nowadays, this is one of the most common techniques used in machine learning, since it provides maximum flexibility and enables computer programs find patterns from the given data [16].

In order to organize this section, and despite their relationship, supervised learning and interpretability related works are presented separately.

### 2.1. Supervised Learning in Retail

Supervised learning models can be grouped in classification and regression models, depending on the type of the target variables *Y*. *Y* are discrete variables in classification models; by contrast, *Y* are continuous variables in regression models. In our case, *Y* are continuous variables that are represented as quantities and time series, so we are focusing on regression models for store foot traffic, conversion rates, and sales forecasts.

In particular, forecasting foot traffic and sales can be considered time series forecasting problems. A time series is a set of observations, where each one is recorded at a specific time [17]. Time series forecasting algorithms have been applied in many areas, e.g., financial market prediction, weather forecasting, and machine prognosis. Retail is one of those areas [6,9,10,18].

Foot traffic and sales forecasting are of significant interest for retailers. For example, Lam et al. [6] highlight the importance of store traffic forecasting and propose a model for the optimization of store labor schedules based on expected traffic. Their model sets store sales potential as a function of store traffic volume, customer type, and customer response to sales force availability. This model first uses the log-transformation of the traffic variable to induce a constant variance, and then a difference with one week lag is applied to this transformed variable. Finally, autoregressive integrated moving average (ARIMA) components are used to adjust for auto-correlations in the residuals. This model was used for a two week ahead forecast and obtained a percentage-forecast error of about 30% on average.

Sales and conversion tickets are commonly modeled as continuous variables and predicted while using regression models, e.g., Yu et al. [8] implemented a Support Vector Regression (SVR) to forecast newspaper/magazine sales. They found out that demographic characteristics, such as gender, age, income, education, and occupation distributions, affect newspaper/magazine sales. However, the relevance of these characteristics is different for other kind of products. Arunraj and Ahrens [7] focused on predicting daily food sales; they used a seasonal autoregressive integrated moving average with external variables (SARIMAX) model, and they concluded that sales forecasts were influenced by external variables, such as day-of-the-week seasonality, month-of-the year seasonality, holidays, festivals, price reductions, and weather. Variations of influence variables among different kinds of products implied that the models should be trained with data dis-aggregated by product or store.

ARIMA is the most popular model to predict foot traffic, showing its effectiveness in many use cases [6,19,20,21]. However, it lacks the modeling of non-linear relationships. Even though it does not perform well in some problems, the ARIMA model is typically used as a baseline model to show the performance of other methods. Cortez et al. [9] used data that were collected in a period of six months from a facial recognition camera to detect the number of daily visitors along with their gender in a sports store. They compared six approaches to predict daily store traffic, using ARIMA as one of their baseline methods, in three different cases. They only detected male visitors, only female and all visitors, forecasting up to a week ahead. Their input vector included the time of the event, special daily event (weekend or holiday; major sports or entertainment event), and weather conditions, such as maximum wind speed, temperature, humidity, and rain. They proposed two metrics to test the performance of the models in this problem: the Average Normalized Mean Absolute Error (ANMAE) and a novel metric named the Average Estimated Store Benefit (AESB). In the AESB metric, the manager of the store executes a forecast for *N* days ahead. Subsequently, an alternative plan is set with better management (e.g., promotions to bring more traffic). Each daily error is multiplied by an average cost that is related to a missed opportunity. Overall Cortez et al. [9] showed that the Support Vector Regression (SVR) was the best forecasting method, regardless of the visitors’ gender and only using time as its input. They used a two-stage approach to obtain optimal performance; first, they used a sensitivity analysis proposed in [22] to discard the least relevant time lag and then they undertook a grid search to find the optimal hyper-parameters.

The previously mentioned method, SVR, is another model that is widely used in time series prediction. SVR is a machine learning model that implements the structural risk minimization inductive principle to obtain a good generalization on a limited number of learning patterns [23]. With a training set L, the goal of the method is to predict the outcome value $\hat{Y}$ of every element of the training set that has, at most, ϵ deviation from the real target variable *Y* [24]. A prediction is calculated as:

(1) $$\hat{Y}\left(X\right)=K\left(w,X\right)+b$$

where *w* is a vector in the same dimensional space as *X* and *b* is a constant, and *K* is a kernel function. Two types of SVR are typically used. The first is linear SVR, which uses a SVR with a lineal kernel, where the kernel function is the dot product. The second is the non-linear SVR with a Gaussian Radial Basis function (RBF) kernel that is defined as:

(2) $$K\left(w,X\right)=e^{-\frac{\left\|X-w\right\|}{2\sigma^{2}}}$$

The predictions need to be as flat as possible. This means that *w* needs to be as small as possible. One way to ensure this is to minimize the norm of *w*. However, this optimization is sometimes not feasible or errors need to be allowed. Vapnik [25] suggested the formulation of the optimization problem where the constant C>0 determines the trade-off between the flatness of the mapping function and the amount up to which the deviation larger than ϵ is tolerated.

K-Nearest Neighbors (KNN) Regression is another non-parametric time series forecasting model [26]. It uses the set of *K* observations that are closest to an input variable *X* to compute a prediction $\hat{Y}$. Specifically, the KNN model matches *X* with the average of its k-nearest neighbors in the training set L. The closeness of a point is evaluated by a distance metric ||·||. An improved version of this method is called Weighted Nearest Neighbors (WKNN), which strongly motivated the creation of the new regression method that is presented in this work. The method uses a weighted average, where the weight of each neighbor is proportional to its proximity [27,28]. It also uses a weighted distance to compute the nearest neighbors. The weight vector is learned during the training phase while using gradient descent (Section 3.2).

Abrishami et al. [10] used data from 56 stores of different business, such as gyms, coffee shops, restaurants, and bars. These data were collected using wireless access points and it was used to forecast the store traffic one week ahead using a Random Forest (RF) and SVR model. This work, unlike [9,18], discarded the use of weather data, because no correlation was observed between traffic data and weather; moreover, its forecasting inaccuracy only added error to the prediction. They used the time of the event as an input vector, including holiday status, special event status, and location (e.g. closeness to schools and being a tourist city). As performance metrics, the Root Mean Squared Error (RMSE) was used, which is the difference between the estimated values and the actual value, as shown in Equation (3).

(3) $$RMSE\left(Y,\hat{Y}\right)=\sqrt{\frac{\sum_{i=1}^{n}{\left(y_{i}-\hat{y_{i}}\right)}^{2}}{n}}$$

where *Y* is the vector of the true target variables and $\hat{Y}$ is the vector of its predicted values, both being vectors of size *n*. The effect of each difference is proportional to the size of the squared error; thus, larger errors have a disproportionately large effect on RMSE. Consequently, RMSE is sensitive to outliers.

The Mean Absolute Error (MAE) was another used measure, which computes the average absolute difference between the predicted values $\hat{y}$ and the real value *y*, as shown in Equation (4).

(4) $$MAE\left(Y,\hat{Y}\right)=\frac{1}{n}\sum_{i=1}^{n}\left|\hat{y_{i}}-y_{i}\right|$$

where $\hat{y}$ and *y* are vectors of size *n*. The Mean Absolute Percentage Error (MAPE) is used for comparison between different datasets. Across all different businesses, the best results were obtained by the SVM model. Afterwards, the authors deepened their study in another work where they tested the performance of Long short-term memory (LSTM). The LSTM model obtained better performance than their previous work. In this new study, the feature vector consisted of a sequence of length 8, which contained the daily foot traffic from the same day in the previous eight weeks. The forecast window used was one month, but, as they used the data of the previous week, it was not possible to use their same feature vector in a real world setting.

In this work, we also analyzed the implementation of a Gaussian Process (GP) [29], which is a collection of random variables, where a finite number has a joint Gaussian distribution. A GP is completely defined by a mean m(x) and co-variance function $K(x,x^{\prime })$ (y∼GP(m,K)). Let us consider a training set L with *X* a set of inputs and *Y* the set of outputs, and a validation set with an input $X_{*}$ and output $Y_{*}$. Because the key assumption in GP is that the data are represented by a Gaussian distribution, the data can be represented as:

(5) $$\left[\begin{array}{c} Y\\ Y_{*} \end{array}\right]=N\left(\left[\begin{array}{c} \mu \\ \mu_{*} \end{array}\right],\left[\begin{array}{cc} \Sigma & \Sigma_{*}\\ \Sigma_{*}^{T} & \Sigma_{**} \end{array}\right]\right)$$

where μ and $\mu_{*}$ are the mean values of the training and validation set. Σ, $\Sigma_{*}$, and $\Sigma_{**}$ are the covariance of the training, training-validation, and validation sets. Y is known, so the target is to compute the value for a new observation $Y_{*}$ given *Y*.

In order to look for a flexible solution, we tested Random Forest [30], which is an ensemble method, a method that uses multiple learning algorithms to obtain the best performance. RF is a combination of random trees (RT), where each tree depends on the value of a random vector sampled independently and with the same distribution for all trees in the forest. The output is computed as the average of each tree output. Each tree is grown and not pruned based on any error measure. This means that the variance of each one of these individual trees is high. However, by averaging the results, this variance can be reduced without increasing the bias.

Finally, we evaluated the Recurrent Neural Network (RNN) [31], which is a type of deep neural network that is specifically designed for sequence modeling.

### 2.2. Interpretable Regressions

The problem that we had testing the algorithms was, in some cases, the performance, but most meaningful to our context, was the interpretability of the results.

In this work, we will understand interpretability as the ability of a model to explain or present its results in understandable terms to a human being [32]. The interpretability depends on the method transparency, which connotes some sense of understanding of the inner mechanisms. Lipton [33] considered transparency at three levels. First at the level of the entire method (simulatability), at the second level individual components, such as parameters (decomposability), and, lastly, at the third level of the training algorithm (algorithmic transparency).

From previous research [5,34,35], we realized that the Dempster–Shafer theory [4] provides interpretability at the third level, as previously indicated. The Dempster–Shafer theory, which is also known as the theory of belief function, is defined, as follows:

Let *X* be the set of all possible states of a system called frame of discernment. A mass assignment function *m* is a function satisfying:

(6) $$m:2^{X}\to \left[0,1\right],\;\;\;m\left(\phi \right)=0,\;\;\;\sum_{A\subseteq X}m\left(A\right)=1$$

where $2^{X}$ is the combination of all possible states, ϕ is the null state, and *A* is each one of the states. The term m(A) can be interpreted as the probability of getting precisely the outcome *A*, and not a subset of *A*. Multiple evidence sources that are expressed by their mass assignment functions of the same frame of discernment can be combined using the Dempster Rule (DR). Given two mass assignment functions $m_{1}$ and $m_{2}$, a new mass assignment function $m_{c}$ can be constructed by the combination of the other two while using the following formula:

(7) $$\begin{array}{cc} m_{c}\left(A\right) & =m_{1}\left(A\right)⊕m_{2}\left(A\right)=\frac{1}{1-K}\sum_{B\cap C=A\neq \phi }m_{1}\left(B\right)m_{2}\left(C\right) \end{array}$$

where *K* is a constant representing the degree of conflict between $m_{1}$ and $m_{2}$ and it is given by the following expression:

(8) $$K=\sum_{B\cap C=\phi }m_{1}\left(B\right)m_{2}\left(C\right).$$

Petit-Renaud and Denux [3] introduced a regression analysis algorithm that is based on a fuzzy extension of belief functions, called Evidential Regression (EVREG). Given an input vector *X*, they predict a target variable *y* in the form of a collection of evidence associated with a mass of belief. This evidence can be fuzzy sets, numbers, or intervals, which are obtained from a training set that is based on a discount function that takes their distance to the input vector *X* and it is pooled using the Dempster combination rule (Equation (7)). They showed that their methods work better than similar standard regression techniques, such as the k-Nearest Neighbors using the data of a simulated impact of a motorcycle with an obstacle.

In our case, the interpretability will be achieved by obtaining the weights for each component of the input. Achieving interpretability in this way allows for us to identify the most relevant variables for forecasting a certain value. This fact lets a retail store manager better react to sudden unexpected changes of these variables. For example, if the stores have a high weight value for holiday existence, then the sudden appearance of a day "like” a holiday will suddenly affect the performance of those stores.

## 3. Materials and Methods

The goal of this research is to predict the key performance indicators (foot traffic, conversion rate, and store sales). The problem of predicting each one of these retail store indicators can be modelled as a discrete time series forecasting problem. This problem has a set of observations $x_{t}$ made at fixed time intervals (e.g., a day), being recorded at a specific time *t* as input [17]. Accordingly, given a time series $X=\{x_{0},x_{1},\ldots ,x_{t}\}$, the time series $\hat{X}=\left\{x_{t+1},\ldots ,x_{n}\right\}$ with n>t needs to be predicted, with [t+1,n] being the forecast window.

**Figure 1:**
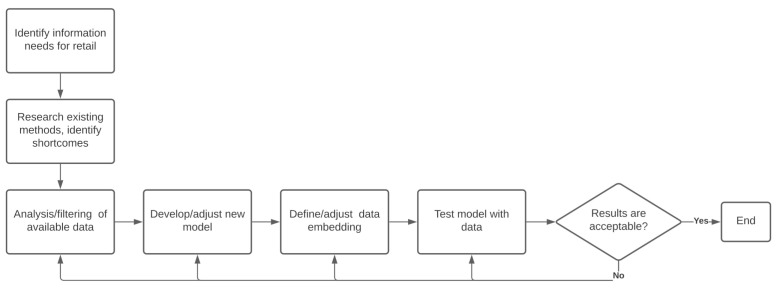
Work methodology.

The methodology for this research work can be summarized, as follows (Figure 1): first, with the participation of experts from the Follow Up company. we identified the information needs that consisted not only of getting a reliable predictor for the three key store performance indicators for the next month, but also obtaining information regarding the main parameters that determine these expected numbers. A month can span from four to six weeks; in this work, a month will span exactly four-weeks, so the forecasting window will be 28 days. Previous works have found that small time windows (e.g., a week) are often applied in real-world settings [9,10,18]; however, store managers need a longer time window than a week in order to carry out a suitable business plan based on expected number of visitors and sales. The next step was to analyze the available data to determine its quality and reliability, and to identify the need for filtering invalid entries. Subsequently, by studying the state of the art of the regression models, we found out that the most accurate methods were the so called black-box models that do not convey more information than the predicted value. This motivated us to develop a new model that is based on an existing one, called EVREG, in order to improve its accuracy and interpretability. Having the data and model, we proceeded with the definition of the embedding vector, after which we could make the first prediction testings. Their results were analyzed in order to adjust/change the data filtering as well as the model and the data embedding until reaching an acceptable result.

The next subsections describe, in more detail, the data acquisition and validation, the development of the regression model, and the data embedding.

### 3.1. Data Acquisition and Validation

The data registered by Follow Up during each hour of operation are the following: the number of visitors (foot traffic), number of tickets, and the total amount of sales (in Chilean currency units) of retail stores. Data on foot traffic are recorded using a three-dimensional (3D) stereoscopic camera that is programmed to recognize, follow, and count people in an enclosed area with the purpose of getting exact knowledge of how crowded the physical space is and the flow of people in it. The cameras have an accuracy of approximately 98% and it can be used both in and outdoors. The ones used specifically for this work were installed in closed retail stores. They are placed at the store entrances and strategic spots in order to capture as much visual information as possible. The way that they detect humans from other static or moving objects is by using video analysis software focused on identifying humans easily in a dynamic environment; this is achieved by using stereoscopic cameras that can make a 3D map of the place and objects moving in it. Once a camera detects people, it starts counting and following them. These cameras are all connected to a private network that allows communication among them and not get ‘confused’ when simultaneously detecting the same person. Data on the number of sales (tickets) and the total amount of sales are obtained directly from the store records. We accessed this data from an Application Programming Interface (API) supplied by Follow Up. The API provides the information on an hourly basis. The obtained data are detailed in Table 1. It should be noted that data in this field are usually proprietary and a valuable asset for the companies. This is the case with Follow Up, which cannot disclose the raw data.

The data are checked for reliability, in particular, a store for which we want to predict the key performance indicators should not have periods where data are missing. Only stores with more than four years of history are selected. Most stores available in the API did not comply with these requirements, so only 20 stores were selected for our experiments, because these ones had the longest history with no null or abnormal values. The extraction window used for the data will be four years, from August 2015 to the same month of 2019. When the stores are selected, the raw data are enriched to produce the input vector used by the method.

**Figure 2:**
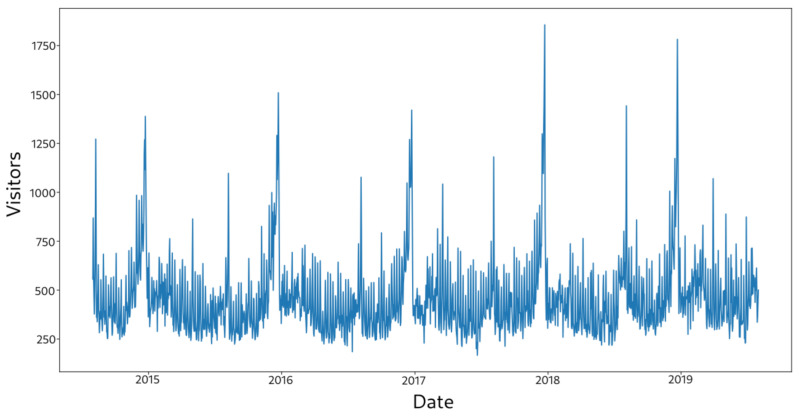
Store visitors by date.

Figure 2 shows an example series of store visitors by hour from August 2015 to August 2019.

### 3.2. Weighted Evidence Regression Model

In this work, we chose to use the Weighted Evidential Regression model (WEVREG) presented by Panay et al. [5]. WEVREG is an interpretable regression method where the prediction of a new observation can be easily followed. This model is based on a previous one that was proposed by Petit-Renaud and Denux [3], which uses the Dempster–Shafer Theory together with a variation of K-Nearest Neighbors (KNN) to produce an evidence-based regression model. WEVREG extends this model by adding weights to each dimension of the attributes making certain parameters have more importance than others when deciding the most similar instances of the KNN process. In addition, this change allows measuring the importance of each attribute in the prediction.

WEVREG uses a distance and a discount function on a new observation to obtain the more similar vectors in a training set, and then it computes the expected value of the observation. Mathematically, the distance function *d* is a weighted euclidean distance that is defined as:

(9) $$d\left(x_{i},x_{j}\right)={\left(\;\sum_{k=1}^{l}{\left|w\cdot \left(x_{ik}-x_{jk}\right)\right|}^{2}\;\right)}^{\frac{1}{2}}$$

where *w* is the weight vector of size *k*, and $x_{ik}$ and $x_{jk}$ are the values of vectors $x_{i}$ and $x_{j}$ at dimension *k*. WEVREG requires that the similarity between vectors be bounded in the [0,1] interval. For this reason, a discount function is applied to the distance function presented. The discount function ϕ between vectors $x_{i}$ and $x_{j}$ using this distance measure will be defined as a Radial Basis Function (RBF):

(10) $$\phi \left(d\left(x_{i},x_{j}\right)\right)=\exp\left(-{\frac{d(x_{i},x_{j})}{\gamma }}^{2}\right)$$

where γ is the radius of the function; intuitively, it defines how far the influence of a vector reaches.

The discount function ϕ will represent the similarity between two vectors (a higher value means higher similarity); this similarity function can be used to compute the mass (influence) of each element in L given an arbitrary vector *x* using the Dempster rule of combination (Equation (7)), obtaining:

(11) $$m_{i}\left(x\right)=\frac{1}{K}\phi \left(d\left(x,x_{i}\right)\right)\prod_{k!=i}\left(1-\phi \left(d\left(x,x_{k}\right)\right)\right)$$

where *K* is a normalization term that is defined by the DST restrictions as:

(12) $$K=\prod_{j=1}^{N}\left(1-\phi \left(d\left(x,x_{i}\right)\right)\right)+\sum_{i=1}^{N}\left[\phi \left(d\left(x,x_{i}\right)\right)\prod_{k!=i}\left(1-\phi \left(d\left(x,x_{k}\right)\right)\right)\right]$$

In the above formulas, the values of the weight vector *w* and value of γ are parameters that are optimized during the training process using gradient descent.

Because of the uncertainty that exists when using DST, there is an additional value $m^{*}$ that must be considered, which is known as the domain mass or the uncertainty of the prediction, and it can be calculated using the following formula

(13) $$m^{*}\left(x\right)=\frac{1}{K}\prod_{i=1}^{N}\left(1-\phi \left(d\left(x,x_{i}\right)\right)\right)$$

Unlike the previous masses that assign a value to a single result, this $m^{*}$ variable assigns a value to the entire range of the domain of *y*. Subsequently, this allows for us choose which value of the domain to use and, thus, obtain different predictions. For example, if we choose the mean value of the range, then we would have the prediction with greater likelihood. Alternatively, if we choose the lowest value of the interval, then we would have the lower bound of the prediction and, if we choose the highest value, then we would have the upper bound. Therefore, in addition to predicting a value, we can deliver a confidence interval of the prediction as a result.

(14) $$\hat{y}=\sum_{i=1}^{N}m_{i}\left(x\right)\cdot y_{j}+m^{*}\left(x\right)\cdot v_{y}\;\;\;\;\;\;v_{y}\in \left[\inf Y,\sup Y\right]$$

The WEVREG method provides two types of interpretability, a global interpretability, which is feature importance obtained by inspecting the values of the weights *w* after they are optimized; and, a local interpretability because the predicted value of a single instance is explained by the attributes of its neighbors; the model can check and evaluate the masses of these neighbors and then identify the ones that are the most similar and their corresponding values.

### 3.3. Embedding

Besides the model chosen for predicting, we need to define the values of the input vectors; this process is known as embedding. Embedding is crucial for achieving good performance, especially in time series regression, where the data can be inputted in many different ways.

Given the importance of embedding, choosing one a *priori* can cause poor results in the prediction. Therefore, four different embedding methods will be tested in this work.

The first vector will consist of the dis-aggregated date features as year, as detailed in Table 2. It is assumed that, in some stores, the day of the week is highly correlated to the expected number of visitors or sales, e.g., a store that is located near offices is expected to receive high number of visitors during work days.

However, the features that were used in Table 2 do not reflect the cyclic quality of time features. For example, the day of the week on the features varies from 0 (Monday) to 6 (Sunday). The furthest day from Sunday is Monday, whereas, in reality, Sunday and Monday are next to each other. Another feature vector will be tested, including these cycles. The new embedding has two features representing the vertical and horizontal components in each feature with a cyclic behaviour. The vector is detailed in Table 3. To build this circular representation, we can use the trigonometric functions sin and cos and thenassign an angle from 0 to 2π to the values for example 0 for Monday and $\frac{6}{7}2\pi$ to Sunday. Note that this transformation effectively captures the cyclical state of these variables, because the distance function previously defined in the model (Equation (9)) is Euclidean and the decomposition into horizontal and vertical components makes contiguous elements have the same distance in the model.

Another variant of these vectors will be tested. For each feature vector presented, a sequence of previously observed values will be added, thus obtaining a total of four feature vectors. These new features will be a sequence of previous time steps of the variable commonly used in time series forecasting. Because the forecast is made with a month window, the values need to have a month of distance. For example, if the visitors for 1 August are being predicted, the previous values will consist of 1 July, 1 June, and so on. The number of months *N* for the feature vector will be set based on the proposed method performance. Table 4 details the new features.

## 4. Experiments and Results

The dataset consists of the number of recorded visitors, the number of sale operations, and the total sales of 20 retail stores from August 2017 to August 2019. Because this is a time series forecasting problem, validation methods, such as random split or cross-validation, cannot be used. The methods performance will be evaluated in a real world setting, with the prediction of a validation set that will consist of one month of data. Thus, data from August 2017 to July 2019 will be treated as a training set, and August 2019 will be the validation set. The Scikt-learn [36], Statsmodels [37], and Pytorch [38] libraries were used for the methods implementation. Scikit-Learn was used for GP, SVR and RF regressors; Statsmodels was used for its SARIMA implementation; Pytorch was used for its implementation of tensor operations and gradient descent. Pytorch was also used for the implementation of the LSTM neural network. Table 5 presents the specifications for the machine and software.

The proposed method (WEVREG) will be compared against techniques previously used in store traffic forecasting. These are SARIMA, SVM-L1 (SVR), RF, GP, and LSTM neural network. Each method will have a single configuration for every store. The configuration for most of the methods was reached using a Grid Search approach, where the values of the parameters in each method are set based on the ones that yield the best performance (lower mean RMSE) while forecasting all stores. The hyper-parameters for the SARIMA method were found using the auto-correlation and partial auto-correlation coefficients. The architecture used for LSTM was as recommended by Abrishami and Kumar [11], with a first layer of LSTM cells and a dense layer with an hyperbolic tangent as an activation function to obtain a single output.

Three measures will be used for assessing the prediction performance of each method. The first one is the RMSE (Equation (3)), another will be MAE (Equation (4)), and the last measure used will be the the Normalized Mean Absolute Error (NMAE), which has previously been used in traffic forecasting. The NMAE between two vectors is calculated as:

(15) $$NMAE\left(Y,\hat{Y}\right)=\frac{\frac{1}{n}\sum_{i=1}^{n}\left|\hat{y_{i}}-y_{i}\right|}{\left|\sup Y-\inf Y\right|}$$

where *Y* and $\hat{Y}$ are vectors of size *n*. NMAE is the MAE normalized by the size of the interval of the real response, as shown in Equation (15).

The performance of each method was calculated for each store using the various embeddings. Subsequently, the mean performance for all stores was computed. The results show the performance of WEVREG in every setting and only the best setting of the compared methods. Table 6, Table 7 and Table 8 show these results.

**Figure 3:**
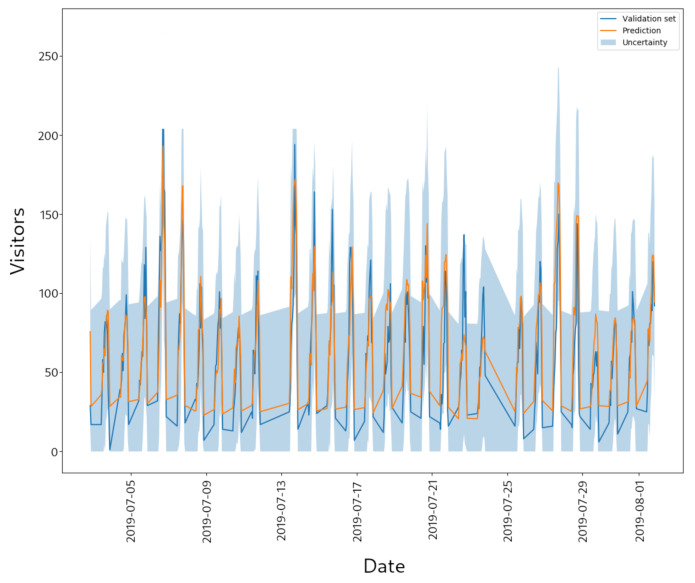
Store visitors prediction.

Figure 3 presents an example of the prediction of the number of visitors for a specific store obtained by the WEVREG method. This figure also shows the confidence interval that the proposed model is able to provide.

**Figure 4:**
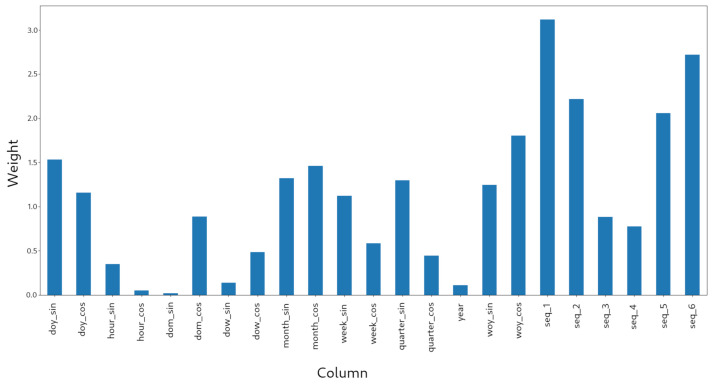
Model weights for the cyclic embedding with the sequence

Figure 4 shows the attribute weights that were obtained after training the WEVREG model using the cyclic embedding. This result illustrates the interpretability that can be obtained from the model, since it is possible to know which attributes are the most important for the prediction and, thus, provide an additional value to the prediction.

We also compare our model to previous ones regarding foot traffic, conversion rate, and sales prediction. Table 9 presents the methods to be compared.

Observing Table 9, it is important to note that the various authors use different data to build their methods, so it is incorrect to perform a quantitative analysis on the models since their results are not comparable. However, we present a qualitative three-dimensional analysis (columns 4–6 in Table 9): the first dimension is the model accuracy, in which we distinguish three levels: high accuracy when the method has reported less than 15 % error, medium accuracy when the error is between 15% and 30%, and low accuracy if the error is greater than 30%. Interpretability is the second dimension: this column shows whether or not the authors present some type of interpretability of their results. Finally, the last dimension for comparison is the confidence interval (CI), which shows whether the method can produce CI in addition to its predictions.

## 5. Discussion

From the analysis of the results that are presented in the previous section, we can see that our approach manages to predict the key retail indicators correctly.

First, we can qualitatively analyze the behavior of the predictions by looking at Figure 3. We can see that, in general, the prediction is well adjusted to the real curve. The method has no problem detecting the peaks and valleys of the real values, although it does not reach the same upper and lower values. In particular, the model hardly reaches the extreme values of the prediction. This shortcoming can be explained by the nature of the prediction with KNN; note that, for predicting a 0 value (the minimum in our case), the model requires that all neighbors it observes must also have value 0; if any of them does not have a 0 value, then it “moves” the prediction towards the center.

Another feature of WEVREG is its ability of provide confidence intervals. In the same Figure, we can observe that almost all the real values are inside the confidence interval. However, we can note that the width of this interval is wide, covering about 30% of the prediction range. This could be because the vectors that are used by the methods after the KNN approach for each prediction are not very similar to one another, obtaining a high uncertainty for the process.

Another remarkable characteristic of the WEVREG model is its interpretability. The model gives the attribute weights after training, as shown in Figure 4. In this case, the model is using the cyclic embedding with the sequence of the six previous months for a particular store. From this figure, it is clear that, for this particular store, some components, such as the year or partial components of the hour and day of the month, are not really important for predicting foot traffic. Additionally, the most important features for predicting foot traffic seem to be the number of visitors observed in previous months. The previous month is the most important, and later observing a decrease in importance followed by an increase in the fifth and sixth month which could be because of the cyclic behavior of the visitors of this particular store.

Talking about performance, WEVREG achieves good performance overall, obtaining comparable results to the best methods that were tested in the literature. Table 6 shows the results for foot traffic predictions; the best model is RF achieving an RMSE of 0.0669, while the next best model is WEVREG using the circular embedding with RMSE of 0.0713. For the case of number of tickets predictions, Table 7 shows a similar behavior. The best two models are GP and RF with RMSE of 0.0712 and 0.0741, respectively. WEVREG using the sequential circular embedding is next reaching RMSE of 0.0795. Finally, Table 8 presents the results for sales predictions. In this case, all of the models, except ARIMA, achieve very similar performance results, the difference of RMSE between the best model and the second to last model is about 0.01; in our case, the best configuration is the normal embedding. Overall, RF obtains the best performance, and this result matches the one previously reported by Abrishami et al. [10]. WEVREG with sequential and circular embedding is the third best model for predicting foot traffic and conversion tickets. For the case of predicting foot traffic and tickets, we can see that WEVREG with circular and sequential embedding is also the third best model. In the case of sales prediction, our model seems to achieve worse performance than the others, but actually all models, except ARIMA, obtain very similar performance values.

Because these are time series problems, it was expected that LSTM, which is a deep-learning based method, would obtain the best results, but it could not outperform our proposed method in general. A possible explanation for this low performance could be the use of a single network architecture for all the stores and the size of the dataset being used. Abrishami and Kumar [11] obtain different results when compared to the presented one using a specific architecture in each tested store.

From the analysis of Table 9, we can see that our method is the only one that is capable of predicting the three indicators at the same time and having high accuracy, having interpretability, and providing confidence interval. We can observe that the rest of the methods may be classified into two groups: those that are derived from ARIMA, which present interpretability and the possibility of having CI, but having medium or low accuracy. The other group is composed by models that aare derived from machine learning methods; they achieve high performance, but do not have interpretability or CI.

In addition, different vector representations were tested in this work, and we split the results of WEVREG to these different embeddings to compare them. The circular embedding is the one yielding the best performance when considering all tested methods. It seems that the breakdown of cyclic features in horizontal and vertical components enables the methods to create a better comprehension of the variables as we anticipated when we introduced this embedding. In particular, for our method, the circular representation with the sequence of previous months is the one yielding best performance in most predicted variables. Another interesting result is that using circular embedding in other methods also implies better performance, even when these methods are not distance-based, which shows that this representation also eases partitioning the feature space for these methods.

## 6. Conclusions

In this work, we present a new interpretable regression model to predict key retail indicators. The results showed that our method achieves levels of precision that are similar to other methods reported in the literature for this same problem. In addition to the prediction methodology, the main contribution of this work is that the proposed model is transparent, which allows for us to discover and analyze the main features that influence the prediction. This allows store managers to obtain knowledge about which are the main variables that influence the behavior of customers, thus allowing them to react to sudden changes in the scenario, before the model adapts itself. Another important contribution that is not present in most previous works, is that it also gives a confidence interval for the predicted values. This method also produces prediction for the three key performance indicators at the same time. These are important implications for practitioners, since these advances are applicable to the stores operation.

As we mentioned earlier, this model can be used to support different processes in the retail business. For example, the foot traffic prediction for a store can be used to plan the number of staff members needed per day. Sales prediction allows for creating more specific sales goals or assigning risk values for meeting these goals. Additionally, these predictions can be used to evaluate the effectiveness of promotions, e.g., by comparing the predicted sales numbers with the actual ones of a promotion campaign.

Because the proposed model is based on KNN, one of its main limitations is that neighbors have to be similar to the scenarios to be predicted in order to generate forecasts with high levels of accuracy. Although a prediction can be made using less similar scenarios, these predictions tend to have high uncertainty. This explains the restriction of having at least four years of data imposed in our study, as explained in Section 3.1. This limitation makes the model unsuitable to operate with new stores that do not have a substantial data history. Another limitation occurs when the model predicts stores located in tourist places. This type of stores drastically change their behavior during vacation periods when compared to the rest of the year. Thus, during regular periods, they have low values and are almost always 0 for the indicators. When the holiday period begins, these indicators increase, sometimes even exceeding those of stores in large cities. We have observed that our model is not capable of predicting this type of behavior, even when the store complies with the constraint of having four years of data.

The proposed model can easily be adapted to any time series regression problem. As mentioned in the introduction, the base model was previously used for predicting health care costs, which is a different type of problem and field from the one that is presented in this work. The main activity that is required to adapt this method to other scenarios would be defining the most convenient embedding for the input variables, since this is one of the most important factors affecting the success of its application.

Another important aspect to mention is the behavior of the model in anomalous situations. A clear example is the COVID-19 pandemic, which caused lockdowns in major cities, forcing retail stores to close or at least to limit the number of customers as stated by Wang et al. [39]. A model, like the proposed one, and probably all models based on historical data, cannot predict this behavior that is generated by external causes. Instead, our model can predict the expected sales for the period of time the store was closed and, thus, these values can be used to estimate the losses that are generated during the period of closure.

Finally, as future work, the model may perhaps be used to create new tools to support the retail processes mentioned above. For instance, it may be possible to build a tool to evaluate the effectiveness of a promotion or another tool suggesting the optimal changes to make to the key indicators in order to achieve a sales goal that is based on the model predictions. Regarding the model itself, future research may study a possible improvement to make the model operate correctly with little data to make it suitable for new stores; a tentative strategy for this purpose may be to cluster similar stores in the training phase and use the ones with the longest history to fulfill the history of new stores. Related to the above, how the different embeddings affect the results when there is little data could also be tested. An embedding that, instead of creating many features, only generates a few ones may perhaps work better for stores with little data. Lastly, another line of future research is to try to improve the interpretability of the model even more. Currently the model elaborates an importance ranking for the features in the prediction; however, it does not report whether these features positively or negatively affect the output. An example of this hypothetical interpretability would be the method showing that sales increase between 14:00 and 18:00 hours, or that foot traffic decreases on Wednesdays. 

## Figures and Tables

**Table 1:** Data obtained from Follow Up Application Programming Interface (API).

Column	Description	Type
store_id	Store identifier.	Integer
time	Time of the event.	Datetime
visitors	Foot traffic in the last hour.	Integer
tickets	The number of tickets in the last hour (number of sales).	Integer
sales	The total sales in the last hour.	Integer

**Table 2:** Time feature vector.

Column	Description
YEAR	Year number
QTR	Quarter number
MON	Month number
WEEK	Week number
WOM	Week of the month
DOY	Day of the year
DOM	Day of the month
DOW	Day of the week
HOUR	Hour of the day

**Table 3:** Cyclic time feature vector.

Column	Description
YEAR	Year number
SIN_QTR	Quarter vertical component
COS_QTR	Quarter horizontal component
SIN_MON	Month vertical component
COS_MON	Month horizontal component
SIN_WEEK	Week vertical component
COS_WEEK	Week horizontal component
SIN_WOM	Week of the month vertical component
COS_WOM	Week of the month horizontal component
SIN_DOY	Day of the year vertical component
COS_DOY	Day of the year horizontal component
SIN_DOM	Day of the month vertical component
COS_DOM	Day of the month horizontal component
SIN_DOW	Day of the week vertical component
COS_DOW	Day of the week horizontal component
SIN_HOUR	Hour of the day vertical component
COS_HOUR	Hour of the day horizontal component

**Table 4:** Sequence features.

Column	Description
$SEQ_{1}$	Indicator value 1 Month before
·	·
·	·
$SEQ_{N}$	Indicator value N months before

**Table 5:** List of software and hardware used in the experiments.

Name	Specification
Processor	Intel(R) Core(TM) i7-8700K @3.70Ghz
Memory	32GB DDR4 3200Mhz
GPU	Nvidia GeForce GTX 1070
Operating System	Archlinux 2019.10.01
Programming Language	Python 3.6
Machine learning libraries	Sklearn 0.23.0 and Statsmodels 0.12.1
Deep learning library	Pytorch 1.7

**Table 6:** Foot traffic prediction performance.

Method	Embedding	RMSE	MAE	NMAE
RF	circular	0.0669±0.02	0.0494±0.01	0.0796±0.02
WEVREG	circular	0.0713±0.02	0.0532±0.02	0.0879±0.03
WEVREG	seq_circular	0.0727±0.02	0.0552±0.01	0.0882±0.02
WEVREG	normal	0.0769±0.02	0.0551±0.01	0.0885±0.02
GP	circular	0.0772±0.02	0.0550±0.01	0.0910±0.02
LSTM	seq_circular	0.0792±0.02	0.0594±0.02	0.0951±0.02
SVM-L1	seq_normal	0.0812±0.02	0.0581±0.01	0.0899±0.02
WEVREG	seq_normal	0.0846±0.02	0.0614±0.01	0.0945±0.02
ARIMA	circular	0.1299±0.14	0.0996±0.14	0.1625±0.23

**Table 7:** Stores tickets prediction performance.

Method	Embedding	RMSE	MAE	NMAE
GP	circular	0.0712±0.01	0.0537±0.01	0.1115±0.02
RF	circular	0.0741±0.01	0.0545±0.01	0.1103±0.02
WEVREG	seq_circular	0.0795±0.01	0.0613±0.01	0.1252±0.02
LSTM	seq_circular	0.0803±0.01	0.0602±0.01	0.1205±0.02
SVM-L1	circular	0.0817±0.01	0.0627±0.01	0.1308±0.02
WEVREG	normal	0.0821±0.02	0.0646±0.01	0.1295±0.03
WEVREG	circular	0.0837±0.01	0.066±0.01	0.1260±0.02
WEVREG	seq_normal	0.0874±0.01	0.0644±0.01	0.1260±0.02
ARIMA	normal	0.1742±0.11	0.1478±0.11	0.2818±0.22

**Table 8:** Stores sales prediction performance.

Method	Embedding	RMSE	MAE	NMAE
RF	circular	0.0668±0.01	0.0477±0.01	0.1130±0.02
SVM-L1	circular	0.0705±0.01	0.055±0.01	0.1248±0.03
LSTM	seq_circular	0.0710±0.01	0.053±0.01	0.1119±0.02
GP	circular	0.0711±0.01	0.0508±0.01	0.1200±0.02
LSTM	circular	0.0731±0.02	0.0575±0.01	0.1304±0.03
WEVREG	normal	0.0757±0.02	0.0592±0.01	0.1334±0.03
WEVREG	seq_normal	0.0759±0.01	0.0576±0.01	0.1225±0.02
WEVREG	circular	0.0761±0.02	0.0588±0.01	0.1289±0.03
WEVREG	seq_circular	0.0770±0.01	0.0585±0.01	0.1255±0.02
ARIMA	normal	0.1147±0.09	0.0881±0.09	0.1889±0.19

**Table 9:** Comparing retail forecasting methods.

Base Model	Authors	Indicator	Accuracy	Interpretable	CI
ARIMA	Lam et al. [6] (1998)	foot traffic	Low	Yes	Yes
SVR	Yu et al. [8] (2013)	sales	High	No	No
SARIMAX	Arunraj and Ahrens [7] (2015)	sales	Medium	Yes	Yes
SVR	Cortez et al. [9] (2016)	foot traffic	High	No	No
Random Forest	Abrishami et al. [10] (2017)	foot traffic	High	No	No
LSTM	Abrishami and Kumar [11] (2018)	foot traffic	High	No	No
WEVREG	Proposed model	foot traffic, sales and conversion rate	High	Yes	Yes

## Data Availability

Raw data cannot be disclosed according to a non-disclosure agreement.
